# Actual status of visitor and telephone restrictions for Japanese psychiatric inpatients: A retrospective observational study

**DOI:** 10.1002/pcn5.70382

**Published:** 2026-08-07

**Authors:** Yoshitaka Saito, Kyo Tomita, Hiroyuki Muraoka, Takaaki Hirooka, Ken Inada

**Affiliations:** ^1^ Department of Psychiatry Kitasato University, School of Medicine Sagamihara Kanagawa Japan

**Keywords:** involuntary admission, physical restraint, seclusion, telephone restriction, visitor restriction

## Abstract

**Aim:**

To clarify the status of visitor and telephone restrictions and the factors associated with these restrictions among patients hospitalized for the treatment of mental disorders.

**Methods:**

This study included 1,998 patients hospitalized for the treatment of mental disorders at the Department of Psychiatry, Kitasato University Hospital, and the Department of Psychiatry, Kitasato University East Hospital between January 1, 2014 and December 31, 2021. The visitor and telephone restriction ratios of the participants were calculated, and their associations with demographic and clinical data and psychiatric diagnosis were comparatively examined.

**Results:**

Of the 1998 participants, 434 (21.7%) experienced visitor restrictions and 631 (31.6%) experienced telephone restrictions. Multivariate logistic regression analysis suggested that young age, involuntary admission, seclusion, physical restraint, schizophrenia spectrum disorders, and manic episodes of bipolar I disorder were associated with visitor restrictions. Additionally, young age, hospitalization days, involuntary admission, seclusion, physical restraint, and manic episodes of bipolar I disorder were associated with telephone restrictions.

**Conclusions:**

The visitor and telephone restriction ratios were 21.7% and 31.6%, respectively. Age, involuntary admission, seclusion, physical restraint, schizophrenia spectrum disorders, and manic episodes of bipolar I disorder were associated with visitor restrictions, and age, hospitalization days, involuntary admission, seclusion, physical restraint, and manic episodes of bipolar I disorder were associated with telephone restrictions. Further research is required to clarify the factors related to visitor and telephone restrictions and their impact on patients to minimize their use.

## INTRODUCTION

In the treatment of inpatients with mental disorders, behavioral restrictions may be necessary for those with severe symptoms. In Japan, such restrictions are regulated by law,^1^ which states that “the administrator of a psychiatric hospital may impose necessary restrictions on the behavior of an inpatient to the extent indispensable for their medical care or protection.” Behavioral restrictions are broadly classified into “seclusion and physical restraint” and “restriction of visitors and communications.” Among these, the law mandates that the use of seclusion and physical restraint be determined based on an examination by a Designated Mental Health Physician.^1^ As seclusion and physical restraint impose a significant burden on patients and are associated with numerous physical and mental adverse events, medical institutions in Japan are required to establish a committee to minimize behavioral restrictions, and efforts are being made to minimize their use. Furthermore, as seclusion and physical restraints constitute major limitations of human rights, investigations into their actual status are continuously being conducted.^2^

Japanese law stipulates that visits and telephone calls with officials from administrative agencies involved in human rights advocacy and lawyers serving as patient representatives cannot be restricted.^1^ However, calls and visits with other parties can be restricted “if there are reasonable grounds, such as causing deterioration of the medical condition or hindering therapeutic effects, to the extent indispensable for medical care and protection.”^1^ The decision to impose these communication and visitation restrictions does not require qualification as a Designated Mental Health Physician and can be implemented under the physician's orders. Visitor restrictions in general hospital wards, intensive care units, and older adult care facilities may adversely affect patients' mental health.^3^, ^4^, ^5^, ^6^ Furthermore, the beneficial effects of visits from family members and supporters have been reported.^7^ Patients with mental disorders consider interactions with the outside world, such as through visits, to be beneficial,^8^ suggesting that free communication and visits may have positive effects on patients' psychiatric symptoms. Although previous studies have suggested these potential benefits, the law permits restrictions based on concerns that communication and visitation may negatively affect psychiatric symptoms. However, no investigations have been conducted on the actual extent of visitor and telephone restrictions. Therefore, this study conducted an exploratory investigation to clarify the status of visitors and telephone restrictions and the factors associated with these restrictions among patients hospitalized for the treatment of mental disorders.

## METHODS

### Participants

The participants of this study were patients hospitalized for the treatment of mental disorders at the Departments of Psychiatry at Kitasato University Hospital and Kitasato University East Hospital between January 1, 2014 and December 31, 2021. The psychiatric ward at Kitasato University East Hospital, which was the subject of this study, was a fully closed ward with 110 beds. Kitasato University East Hospital closed at the end of March 2020, and its psychiatric services were transferred to Kitasato University Hospital in April 2020. The psychiatric ward at Kitasato University Hospital has 42 beds and is a fully closed ward. An opt‐out procedure was implemented to ensure the opportunity to refuse participation; patients who did not refuse participation were included in this study. A total of 1,998 individuals were included in this study. If the same patient was hospitalized multiple times during the data collection period, each hospitalization was treated separately.

This study was approved by the Ethics Committee of Kitasato University Hospital (approval no. B25‐033, date of approval: June 23, 2025) and conducted in accordance with the ethical standards of the Declaration of Helsinki.

### Data collection

We investigated the presence or absence of visitor and telephone restrictions among the participants. Additionally, to evaluate factors related to visitor and telephone restrictions, we examined demographic data (age, sex, number of psychiatric hospitalizations, hospital admission type [voluntary or involuntary]), psychiatric diagnosis, and clinical data (hospitalization days, presence or absence of seclusion during hospitalization, and presence or absence of physical restraints during hospitalization). In this study, diagnoses were classified based on the *Diagnostic and Statistical Manual of Mental Disorders*, 5th edition.^9^

### Definition of visitor and telephone restriction

Visitor restriction was defined as one or more days during the hospitalization period when visits were restricted by a physician's order. At both sites, inward visits were prohibited from April 2020 onwards as a countermeasure against COVID‐19 infection, and visits were conducted in a separate room. Therefore, from April 2020 onwards, patients were treated as having visitor restrictions if there was at least 1 day during the hospitalization period when visits in a separate room were restricted by a physician's order.

Furthermore, at both sites, calls using mobile phones were prohibited for all patients during the study period; calls were only permitted via public telephones installed in the wards. Communication with family and acquaintances via email or social networking services using mobile phones was permitted in a separate room for approximately 10 min per session, but could be restricted at the physician's discretion, depending on the patient's condition. In this study, telephone restriction was defined as having at least 1 day during the hospitalization period when either the use of public telephones or email/social networking services on mobile phones was restricted by a physician's order.

### Statistical analysis

Participants were classified into two groups based on the presence or absence of visitor restrictions, and age, hospitalization days, and number of psychiatric hospitalizations were compared using the Mann–Whitney *U* test. For participants with visitor restrictions, we calculated the percentage of hospitalization days with restriction. Furthermore, participants were classified into two groups according to sex, hospital admission type at the time of admission, presence or absence of seclusion during hospitalization, presence or absence of physical restraints during hospitalization, and psychiatric diagnosis. The visitor restriction ratio for each group was calculated and compared using the chi‐square test. In addition, considering the possible impact of the COVID‐19 pandemic on restrictions, a sensitivity analysis was performed, which was limited to participants whose discharge date was on or before December 31, 2019.

Similarly, participants were classified into two groups based on the presence or absence of telephone restrictions, and age, hospitalization days, and number of psychiatric hospitalizations were compared using the Mann–Whitney *U* test. For participants with telephone restrictions, we calculated the percentage of hospitalization days with restriction. Furthermore, participants were classified into two groups according to sex, hospital admission type at the time of admission, presence or absence of seclusion during hospitalization, presence or absence of physical restraints during hospitalization, and psychiatric diagnosis. The telephone restriction ratio for each group was calculated and compared using the chi‐square test. In addition, considering the possible impact of the COVID‐19 pandemic on restrictions, a sensitivity analysis was performed, which was limited to participants whose discharge date was on or before December 31, 2019. In the primary analysis of this study, we performed 38 *χ* ^2^ tests and 6 Mann–Whitney *U* tests, for a total of 44 tests. Including the sensitivity analysis, we performed a total of 88 tests throughout this study. Therefore, the significance level of 5% was set at 5.7 × 10^−4^ (0.05/88) using the Bonferroni correction for multiple testing with 88 tests.

In addition, multivariate logistic regression analyses were performed with visitor and telephone restriction as the dependent variable. The items for which statistically significant differences were found in the *χ* ^2^ tests and Mann–Whitney *U* tests were set as independent variables. With regard to bipolar and related disorders, we used the subtypes of manic episode or depressive episode as independent variables. Furthermore, patients with seclusion or physical restraint during hospitalization may have been subject to visitor and telephone restrictions not only due to clinical decision but also because of structural consequence. It is assumed that patients with seclusion or physical restraint during hospitalization have a higher severity of illness. To account for these factors, we excluded participants with seclusion or physical restraint during hospitalization and performed an additional multivariate logistic regression analysis as a sensitivity analysis, with visitor and telephone restrictions as dependent variables. For the multivariate model, statistical significance was set at the standard *p* < 0.05, as the analysis evaluates the simultaneous, adjusted effects of variables within a single model. For the binary data of the independent variables, “present” was coded as 1 and “absent” as 0. All statistical analyses were performed using SPSS Statistics 26.0 (IBM).

## RESULTS

**Figure 1:**
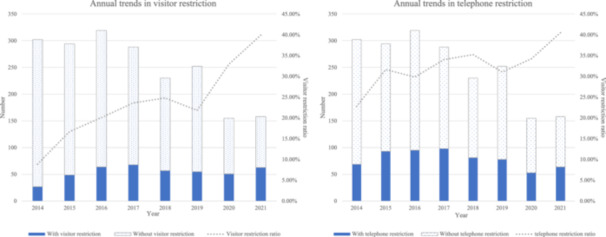
The overall visitor and telephone restriction ratios for all participants were 21.7% and 31.6%, respectively. In 2014, the visitor and telephone restriction ratios were 8.9% and 22.8%, respectively, whereas in 2021, the visitor and telephone restriction ratios were 39.9% and 40.5%, respectively; both increased from 2014 to 2021.

**Figure 2:**
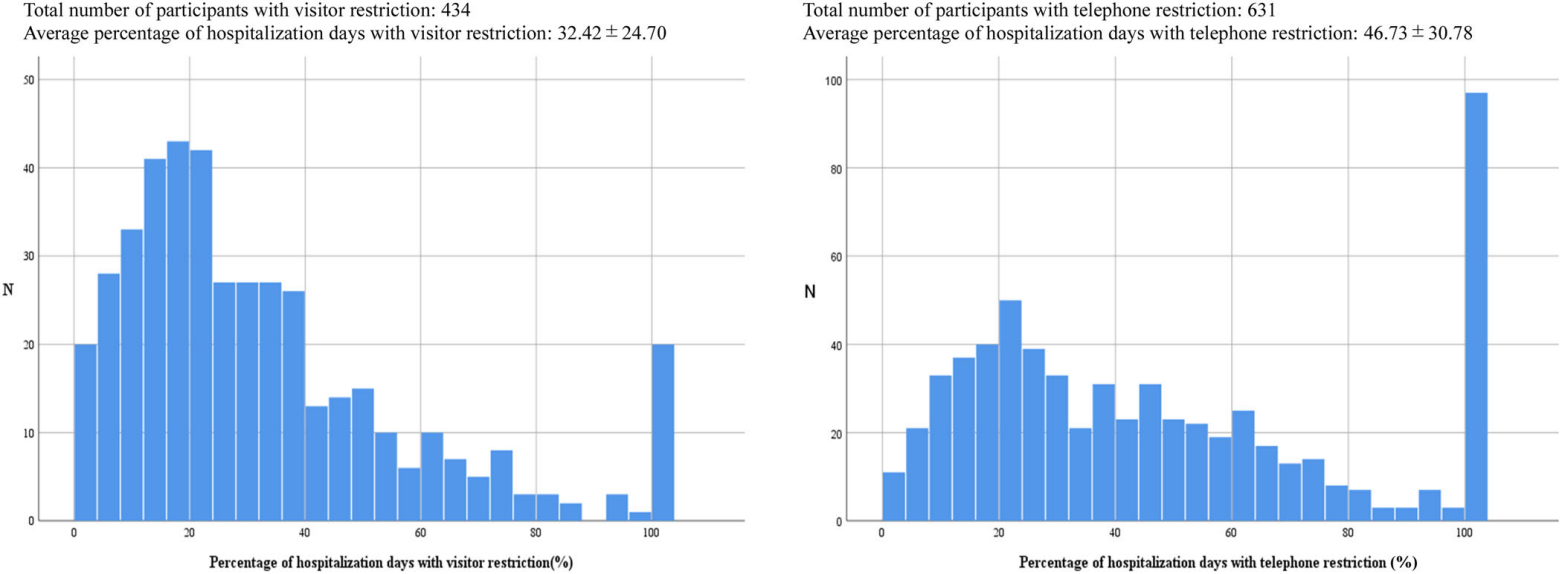
While the distributions for both visitor restrictions and telephone restrictions peaked at around 20% of the total hospital stay, a peak was also observed among participants with restrictions for the entire duration of their hospital stay.

The visitor and telephone restriction ratios, demographic data, clinical data, and psychiatric diagnoses of all participants are shown in Table 1. The annual trends in visitor and telephone restriction ratios are shown in Figure 1 and Supporting information S1: Table S1, respectively. The overall visitor and telephone restriction ratios for all participants were 21.7% and 31.6%, respectively. In 2014, the visitor and telephone restriction ratios were 8.9% and 22.8%, respectively, whereas in 2021, the visitor and telephone restriction ratios were 39.9% and 40.5%, respectively; both increased from 2014 to 2021. Figure 2 shows a histogram depicting the percentage of hospitalization days with visitor and telephone restriction for the participants with restriction. While the distributions for both visitor restrictions and telephone restrictions peaked at around 20% of the total hospital stay, a peak was also observed among participants with restrictions for the entire duration of their hospital stay.

**Table 1:** Demographic and clinical data.

	Psychiatric inpatients (*N* = 1998)
Age, mean ± SD	49.85 ± 17.29
Sex (female), *n* (%)	1351 (67.6)
Visitor restriction, *n* (%)	434 (21.7)
Telephone restriction, *n* (%)	631 (31.6)
Number of psychiatric hospitalizations, mean ± SD	3.86 ± 4.11
Hospital admission type (involuntary admission), *n* (%)	1269 (63.5)
Hospitalization days, mean ± SD	74.00 ± 55.06
Seclusion, *n* (%)	376 (18.9)
Physical restraint, *n* (%)	556 (27.8)
Diagnosis (including co‐morbid diagnoses)	
Neurodevelopmental disorders	205 (10.3)
Schizophrenia spectrum and other psychotic disorders	855 (42.8)
Bipolar and related disorders	158 (7.9)
Bipolar I disorder manic episode	63 (3.2)
Bipolar I disorder depressive episode	81 (4.1)
Depressive disorders	428 (21.4)
Anxiety disorders	51 (2.6)
Obsessive‐compulsive and related disorders	33 (1.7)
Trauma and stressor‐related disorders	31 (1.6)
Dissociative disorders	88 (4.4)
Somatic symptoms and related disorders	51 (2.6)
Feeding and eating disorders	56 (2.8)
Substance‐related and addictive disorders	104 (5.2)
Neurocognitive disorders	68 (3.4)
Personality disorders	77 (3.9)

The associations between demographic and clinical data and visitor and telephone restrictions are shown in Tables 2 and 3, respectively. In the group with visitor restrictions, the mean age was significantly younger, and the duration of hospitalization was significantly longer than those in the group without visitor restrictions. Additionally, the visitor restriction ratio was significantly higher in males than in females, in patients with involuntary admission than in those with voluntary admission, in patients with seclusion during hospitalization than in those without, and in patients with physical restraints during hospitalization than in those without.

**Table 2:** Association between demographic and clinical data and visitor restriction.

Demographic and clinical data	With visitor restriction	Without visitor restriction	All	*p*‐value
Age, mean ± SD	45.71 ± 15.54	51.00 ± 17.57	49.85 ± 17.29	5.0 × 10^−8^ *
Hospitalization days, mean ± SD	84.97 ± 61.17	70.96 ± 52.86	74.00 ± 55.06	2.0 × 10^−6^ *
Number of psychiatric hospitalizations, mean ± SD	3.61 ± 3.98	3.92 ± 4.14	3.86 ± 4.11	0.05

		Visitor restriction ratio, *n* (%)	*χ* ^2^ (*df* = 1)	*p*‐value
Sex	Female (*n* = 1351)	248 (18.4)	27.78	1.4 × 10^−7^ *
	Male (*n* = 647)	186 (28.7)		
Hospital admission type	Involuntary (*n* = 1269)	416 (32.8)	250.21	2.3 × 10^−56^ *
	Voluntary (*n* = 729)	18 (2.5)		
Seclusion during hospitalization	Yes (*n* = 376)	292 (77.7)	852.33	2.3 × 10^−187^ *
	No (*n* = 1622)	142 (8.8)		
Physical restraint during hospitalization	Yes (*n* = 566)	230 (40.6)	166.16	5.1 × 10^−38^ *
	No (*n* = 1432)	204 (14.2)		

*Note*: As the level of significance (*p* < 5.7 × 10^−4^) was within the 5% significance level, based on the Bonferroni correction, it was considered in the multiplicity of the tests.

* *p* < 0.05, after Bonferroni correction.

**Table 3:** Association between demographic and clinical data and telephone restriction.

Demographic and clinical data	With telephone restriction	Without telephone restriction	All	*p*‐value
Age, mean ± SD	46.70 ± 16.47	51.31 ± 17.47	49.85 ± 17.29	3.8 × 10^−8^ *
Hospitalization days, mean ± SD	85.22 ± 61.37	68.82 ± 51.09	74.00 ± 55.06	1.2 × 10^−8^ *
Number of psychiatric hospitalizations, mean ± SD	3.65 ± 3.90	3.95 ± 4.20	3.86 ± 4.11	0.17

		Telephone restriction ratio, *n* (%)	*χ* ^2^ (*df* = 1)	*p*‐value
Sex	Female (*n* = 1351)	378 (28.0)	25.06	5.6 × 10^−7^ *
	Male (*n* = 647)	253 (39.1)		
Hospital admission type	Involuntary (*n *= 1269)	606 (47.8)	421.00	1.5 × 10^−93^ *
	Voluntary (*n* = 729)	25 (3.4)		
Seclusion during hospitalization	Yes (*n* = 376)	334 (88.8)	702.51	8.5 × 10^−155^ *
	No (*n* = 1622)	297 (18.3)		
Physical restraint during hospitalization	Yes (*n* = 556)	354 (62.5)	350.38	3.5 × 10^−78^ *
	No (*n* = 1432)	277 (19.3)		

*Note*: As the level of significance (*p* < 5.7 × 10^−4^) was within the 5% significance level, based on the Bonferroni correction, it was considered in the multiplicity of the tests.

* *p* < 0.05, after Bonferroni correction.

Similarly, in the group with telephone restrictions, the age was significantly lower, and the duration of hospitalization was significantly longer than in the group without telephone restrictions. Additionally, the telephone restriction ratio was significantly higher in males than in females, in patients with involuntary admissions than in those with voluntary admissions, in patients with seclusion during hospitalization than in those without, and in patients with physical restraints during hospitalization than in those without.

The associations between psychiatric diagnoses and visitor and telephone restrictions are presented in Tables 4 and 5. Patients with schizophrenia spectrum and other psychotic disorders and those with bipolar and related disorders had significantly higher visitor and telephone restriction ratios than those without these diagnoses. Among bipolar and related disorders, both ratios were significantly higher for manic episodes in bipolar I disorder, but no significant difference was observed for depressive episodes in bipolar I disorder. Furthermore, for depressive disorders, both ratios were significantly lower than those without depressive disorders.

**Table 4:** Association between psychiatric diagnosis and visitor restriction.

Diagnosis (including comorbid diagnoses)		Visitor restriction ratio, *n* (%)	*χ* ^2^ (*df* = 1)	*p*‐value
Schizophrenia spectrum and other psychotic disorders	Yes (*n* = 855)	265 (31.0)	75.57	3.5 × 10^−18^ *
	No (n = 1143)	169 (14.8)		
Bipolar and related disorders	Yes (*n* = 158)	52 (32.9)	12.63	3.8 × 10^−4^ *
	No (*n* = 1840)	382 (20.8)		
Bipolar I disorder manic episode	Yes (*n *= 63)	37 (58.7)	52.40	4.5 × 10^−13^ *
	No (*n* = 1935)	397 (20.5)		
Bipolar I disorder depressive episode	Yes (*n* = 81)	10 (12.3)	4.37	0.04
	No (*n* = 1917)	424 (22.1)		
Depressive disorders	Yes (*n* = 428)	42 (9.8)	45.43	1.6 × 10^−11^ *
	No (*n* = 1570)	392 (25.0)		
Anxiety disorders	Yes (*n* = 51)	3 (5.9)	7.72	5.5 × 10^−3^
	No (*n* = 1947)	431 (22.1)		
Obsessive‐compulsive and related disorders	Yes (*n* = 33)	2 (6.1)	4.84	0.03
	No (*n* = 1965)	432 (22.0)		
Trauma and stressor‐related disorders	Yes (*n* = 31)	1 (3.2)	6.34	0.01
	No (*n* = 1967)	433 (22.0)		
Dissociative disorders	Yes (*n* = 88)	18 (20.5)	0.09	0.77
	No (*n* = 1910)	416 (21.8)		
Somatic symptoms and related disorders	Yes (*n* = 51)	7 (13.7)	1.97	0.16
	No (*n* = 1947)	427 (21.9)		
Feeding and eating disorders	Yes (*n* = 56)	7 (12.5)	2.88	0.09
	No (*n* = 1942)	427 (22.0)		
Neurodevelopmental disorders	Yes (*n* = 205)	30 (14.6)	6.75	0.01
	No (*n* = 1793)	404 (22.5)		
Substance‐related and addictive disorders	Yes (*n* = 104)	23 (22.1)	0.01	0.92
	No (*n* = 1894)	411 (21.7)		
Neurocognitive disorders	Yes (*n *= 68)	7 (10.3)	5.41	0.02
	No (*n* = 1930)	427 (22.1)		
Personality disorders	Yes (*n *= 77)	17 (22.1)	0.006	0.94
	No (*n* = 1921)	417 (21.7)		

*Note*: As the level of significance (*p* < 5.7 × 10^−4^) was within the 5% significance level, based on the Bonferroni correction, it was considered in the multiplicity of the tests.

* *p* < 0.05, after Bonferroni correction.

**Table 5:** Association between psychiatric diagnosis and telephone restriction.

Diagnosis (including comorbid diagnoses)		Telephone restriction ratio, *n* (%)	*χ* ^2^ (*df* = 1)	*p*‐value
Schizophrenia spectrum and other psychotic disorders	Yes (*n* = 855)	356 (41.6)	69.94	6.1 × 10^−17^ *
	No (*n* = 1143)	275 (24.1)		
Bipolar and related disorders	Yes (*n* = 158)	63 (39.9)	5.46	0.02
	No (*n* = 1840)	568 (30.9)		
Bipolar I disorder manic episode	Yes (*n* = 63)	44 (69.8)	44.07	3.2 × 10^−11^ *
	No (*n* = 1935)	587 (30.3)		
Bipolar I disorder depressive episode	Yes (*n* = 81)	14 (17.3)	7.99	4.7 × 10^−3^
	No (*n* = 1917)	617 (32.2)		
Depressive disorders	Yes (*n* = 428)	68 (15.9)	62.09	3.3 × 10^−15^ *
	No (*n* = 1570)	563 (35.9)		
Anxiety disorders	Yes (*n* = 51)	6 (11.8)	9.51	2.0 × 10^−3^
	No (*n* = 1947)	625 (32.1)		
Obsessive‐compulsive and related disorders	Yes (*n* = 33)	3 (9.1)	7.86	5.1 × 10^−3^
	No (*n* = 1965)	628 (32.0)		
Trauma and stressor‐related disorders	Yes (*n *= 31)	2 (6.5)	9.20	2.4 × 10^−3^
	No (*n* = 1967)	629 (32.0)		
Dissociative disorders	Yes (*n* = 88)	22 (25.0)	1.85	0.17
	No (*n *= 1910)	609 (31.9)		
Somatic symptoms and related disorders	Yes (*n *= 51)	7 (13.7)	7.72	5.5 × 10^−3^
	No (*n* = 1947)	624 (32.0)		
Feeding and eating disorders	Yes (*n* = 56)	27 (48.2)	7.38	6.6 × 10^−3^
	No (*n* = 1942)	604 (31.1)		
Neurodevelopmental disorders	Yes (*n* = 205)	54 (26.3)	2.90	0.09
	No (*n* = 1793)	577 (32.2)		
Substance‐related and addictive disorders	Yes (*n* = 104)	37 (35.6)	0.81	0.37
	No (*n* = 1894)	594 (31.4)		
Neurocognitive disorders	Yes (*n* = 68)	20 (29.4)	0.15	0.70
	No (*n* = 1930)	611 (31.7)		
Personality disorders	Yes (*n* = 77)	18 (23.4)	2.50	0.11
	No (*n* = 1921)	613 (31.9)		

*Note*: As the level of significance (*p* < 5.7 × 10^−4^) was within the 5% significance level, based on the Bonferroni correction, it was considered in the multiplicity of the tests.

* *p* < 0.05, after Bonferroni correction.

The results of the sensitivity analysis limited to participants whose discharge date was on or before December 31, 2019, are shown in Supporting information S1: Tables S2–S5. The results of the sensitivity analysis were similar to those of the primary analyses.

In addition, Tables 6 and 7 show the results of multivariate logistic regression analyses with visitor and telephone restrictions as dependent variables. Age, involuntary admission, seclusion during hospitalization, physical restraint during hospitalization, schizophrenia spectrum and other psychotic disorders, and bipolar I disorder manic episode were significantly associated with visitor restriction. Furthermore, age, hospitalization days, involuntary admission, seclusion during hospitalization, physical restraint during hospitalization, and bipolar I disorder manic episode were significantly associated with telephone restriction.

**Table 6:** The results of multivariable logistic regression analyses for visitor restriction.

Independent variables	Odds ratio	95% CI		*p*‐value
		Lower limit	Upper Limit	
Age	0.984	0.975	0.994	1.2 × 10^−3^ *
Sex (female)	0.829	0.607	1.132	0.24
Hospitalization days	1.001	0.998	1.003	0.68
Involuntary admission	6.572	3.837	11.25	6.9 × 10^−12^ *
Seclusion during hospitalization	19.74	14.20	27.45	1.7 × 10^−70^ *
Physical restraint during hospitalization	2.857	2.103	3.883	1.9 × 10^−11^ *
Schizophrenia spectrum and other psychotic disorders	1.637	1.150	2.329	6.2 × 10^−3^ *
Bipolar I disorder manic episode	2.770	1.261	6.081	0.01*
Depressive disorders	1.350	0.816	2.236	0.24

* *p* < 0.05.

**Table 7:** The results of multivariable logistic regression analyses for telephone restriction.

Independent variables	Odds ratio	95% CI		*p*‐value
		Lower limit	Upper Limit	
Age	0.983	0.975	0.991	8.0 × 10^−5^ *
Sex (female)	0.847	0.633	1.135	0.27
Hospitalization days	1.003	1.001	1.005	0.01*
Involuntary admission	10.49	6.530	16.84	2.4 × 10^−22^ *
Seclusion during hospitalization	20.56	13.97	30.26	4.3 × 10^−53^ *
Physical restraint during hospitalization	5.366	4.047	7.114	1.8 × 10^−31^ *
Schizophrenia spectrum and other psychotic disorders	1.282	0.938	1.751	0.12
Bipolar I disorder manic episode	2.596	1.097	6.146	0.03*
Depressive disorders	0.860	0.561	1.319	0.49

* *p* < 0.05.

The results of the multivariate logistic regression analysis, conducted after excluding participants with seclusion or physical restraint during hospitalization, are shown in Supporting information S1: Tables S6 and S7. Based on the results of the sensitivity analysis, involuntary admission was significantly associated with visitor restriction. Age, hospitalization days, and involuntary admission were significantly associated with telephone restriction.

## DISCUSSION

This is the first study to clarify the status of visitors and telephone restrictions for psychiatric inpatients in Japan. During hospitalization, 21.7% and 31.6% of the participants were subject to visitor and telephone restrictions, respectively. From 2014 to 2021, both visitor and telephone restrictions showed increasing trends. It is possible that visitor restrictions were more likely to be implemented after 2020 due to the impact of the COVID‐19 pandemic. Furthermore, since the number of hospitalized patients decreased after 2020, the proportion of critically ill patients increased relative to the total, and it may be inferred that a higher proportion of patients were subject to visitor and telephone restrictions. Involuntary admission, seclusion, and physical restraint are increasing in Japan, and debates regarding coercive care in psychiatric medicine concerning human rights are ongoing globally.^10^ These discussions primarily focused on involuntary admission and seclusion/physical restraint, whereas visitor and telephone restrictions, despite being limited to patient rights, have not been sufficiently discussed. Although there are limitations to this single‐center retrospective study, clarifying the actual status of visitor and telephone restrictions is a strength.

In this study, multivariate logistic regression analysis suggested that young age, involuntary admission, seclusion, physical restraint, schizophrenia spectrum disorders, and manic episodes of bipolar I disorder were associated with visitor restrictions. Additionally, young age, hospitalization days, involuntary admission, seclusion, physical restraint, and manic episodes of bipolar I disorder were associated with telephone restrictions. Male sex, involuntary admission, schizophrenia, bipolar disorder, and younger age have been reported as risk factors for violence and aggression among psychiatric inpatients.^11^, ^12^ Additionally, manic episodes, psychotic episodes, involuntary admissions, and clinical severity have been reported as predictors of coercive measures, such as seclusion and physical restraint.^13^, ^14^ Although this study did not directly investigate the reasons for visitor and telephone restrictions, common reasons for such restrictions typically include the risk of violence, suicidal tendencies, interpersonal conflict, or clinical deterioration. Considering these findings, it is likely that visitor and telephone restrictions are more frequently implemented in conditions involving violence or aggression, including in patients requiring seclusion and/or physical restraints.

Japanese psychiatric hospitalizations involve a high proportion of involuntary admissions^15^ and a high frequency of physical restraints.^16^ Japanese psychiatrists justify forced hospitalization by arguing that individuals in mental crises require protection because they cannot make decisions or understand their situations.^17^ In Japan, the proportion and duration of coercive measures are higher than those in other countries,^18^ and the tolerance for coercion may extend to other types and durations.^18^ Given the background of psychiatric care in Japan, patients with involuntary admissions, seclusion, and physical restraints are considered more susceptible to visitor and telephone restrictions.

It should be noted that patients with seclusion or physical restraint during hospitalization may have been subject to visitor and telephone restrictions due to structural factors. In other words, patients with seclusion cannot receive visitors or make phone calls unless they are allowed to leave their isolation rooms, so they automatically receive visitor and telephone restrictions. The same applies to telephone restrictions for patients who were physically restrained. On the other hand, the results of this study indicate that some patients who were isolated or physically restrained did not receive visitor and telephone restrictions. This is likely because, for some patients, clinical instructions were in place to lift isolation or physical restraint during visits or telephone calls.

Since such structural factors may have partially influenced the results, we performed an analysis excluding participants who were subjected to seclusion or physical restraints in this study. In a multivariate logistic regression analysis conducted after excluding subjects who were subjected to seclusion or physical restraints, involuntary admission was significantly associated with visitor restrictions, while involuntary admission, age, and length of hospitalization were significantly associated with telephone restrictions. Since seclusion and physical restraints are generally applied to patients with higher severity of illness, these results are considered to reflect a degree of adjustment for confounding by severity. As involuntary admission remained significantly associated with visitor and telephone restrictions even after excluding participants who underwent seclusion or physical restraints, this suggests that these restrictions may be a form of “less visible coercion” that co‐occurs with more explicit coercive practices. In other words, the results of this study suggest that there may have been cases where visitor and telephone restrictions were routinely imposed simply because patients were admitted involuntarily, regardless of the severity of their condition.

The observed restrictions in this study reflect not only clinical judgments but also systemic and institutional governance. First, periodic government audits under the Act on Mental Health and Welfare mandate strict documentation and protect patient rights, acting as a deterrent against arbitrary restrictions. Second, there is the role of the “Committees for Minimizing Restrictions on Behavior” established at each facility. This internal oversight extends to visitor and telephone restrictions, promoting routine reassessments and early discontinuation. Thus, our data reflects the regulatory impact of these external and internal safeguards. Nevertheless, as demonstrated in this study, many patients are currently subject to visitor and telephone restrictions. To minimize these restrictions, it is necessary to periodically review and revise the initiatives undertaken by these government audits and committees within each facility.

This study demonstrates the actual status of visitors, telephone restrictions, and associated factors. Visits from family members during hospitalization help in recovery from mental illness.^19^. Furthermore, reports have shown an association between family calls, visits, and comprehensive discharge planning.^20^ Studies on the impact of telephone restrictions on patients have not been conducted sufficiently. Considering these reports, there is insufficient evidence to support visitor and telephone restrictions for patient recovery or for early discharge. Nevertheless, the findings revealed that many patients are subject to visitor and telephone restrictions. Further research is needed to clarify the factors related to visitor and telephone restrictions and their impact on patients to minimize their use.

### Limitations

This study had some limitations. First, psychiatric symptom severity was not evaluated using rating scales; therefore, the effect of severity on restrictions was not considered. Second, the content of inpatient treatments, such as pharmacotherapy and electroconvulsive therapy, was not evaluated; therefore, their impact on restrictions was not accounted for. Third, this was a retrospective study conducted at a single center in Japan, which limits generalizability of the results. In particular, the study design includes facility policies that establish baseline levels of communication restrictions (e.g., prohibition of mobile phone calls for all patients, limited access to email/social networking services). Defining “telephone restriction” in light of these institutional factors significantly limits the generalizability of this study. Fourth, if the same patient was hospitalized multiple times during the data collection period, each hospitalization was treated as a separate case. Therefore, multiple registrations of the same patient may have influenced the results. Fifth, it cannot be denied that the COVID‐19 pandemic may have influenced physicians' decisions regarding patient restrictions for data from 2020 onwards. Sixth, the reasons for implementing visitor and telephone restrictions were not investigated. Seventh, since the definitions of visitor and telephone restrictions in this study are based on binary data, there are limitations to the discussion regarding the duration, frequency, and intensity of these restrictions. Based on these limitations, future multicenter prospective studies that include severity and treatment content as evaluation items and assess the reasons for restrictions are necessary.

## CONCLUSION

In this study, multivariate logistic regression analysis suggested that young age, involuntary admission, seclusion, physical restraint, schizophrenia spectrum disorders, and manic episodes of bipolar I disorder were associated with visitor restrictions. Additionally, young age, hospitalization days, involuntary admission, seclusion, physical restraint, and manic episodes of bipolar I disorder were associated with telephone restrictions. However, it is important to note that factors associated with these restrictions may be subject to confounding effects by the severity of mental illness. Further research is needed to clarify the factors related to visitor and telephone restrictions and their impact on patients to minimize their use.

## AUTHOR CONTRIBUTIONS

Yoshitaka Saito was critically involved in collecting and analyzing the data and wrote the first draft of the manuscript. Kyo Tomita, Hiroyuki Muraoka, and Takaaki Hirooka contributed to participant recruitment, data collection, and interpretation. Ken Inada supervised the entire project and was critically involved in the data collection, design, analysis, and interpretation. All authors approved the final version of the manuscript and agreed to be accountable for all aspects of the study.

## CONFLICT OF INTEREST STATEMENT

H. M. has received personal fees from Eisai, Janssen, Lundbeck Japan, Takeda Pharmaceutical Company, Meiji Seika Pharma, Mochida, MSD, Otsuka, Pfizer, Viatris, and Sumitomo Pharma in the last 3 years. K. I. received personal fees from Daiichi Sankyo, Eisai, Eli Lilly, Janssen, Lundbeck Japan, Meiji Seika Pharma, Mitsubishi Tanabe Pharma, Mochida, MSD, Nipro, Novartis, Otsuka, Pfizer, Shionogi, Sumitomo Pharma, Yoshitomiyakuhin, and Viatris, and he received research grant support from Mochida and Sumitomo Pharma. The remaining authors declare no conflicts of interest.

## ETHICS APPROVAL STATEMENT

This study was approved by the Ethics Committee of Kitasato University Hospital (approval no. B25‐033, date of approval: June 23, 2025) and conducted in accordance with the ethical standards of the Declaration of Helsinki. As this was a retrospective observational study using existing medical information, the need for signed informed consent from patients was waived by the Kitasato University Hospital Ethics Committee. Patients were informed about the purpose and procedures of the study and were given the option to opt out or refuse participation.

## PATIENT CONSENT STATEMENT

Not applicable.

## CLINICAL TRIAL REGISTRATION

Not applicable.

## Supporting information

Supporting File 1.

## Data Availability

The data that support the findings of this study are available on request from the corresponding author. The data are not publicly available due to privacy or ethical restrictions. The datasets generated and/or analyzed during the current study are not publicly available for ethical reasons, but are available from the corresponding author upon reasonable request.
