# Supplementary material for: Actual status of visitor and telephone restrictions for Japanese psychiatric inpatients: A retrospective observational study
Source: PCN Rep. 2026 Aug 7;5(3):e70382. doi: 10.1002/pcn5.70382 (PMC13448776; doi:10.1002/pcn5.70382)
Supplement: Supplementary file 1 [file PCN5-5-e70382-s001.docx]

| Table S1. Annual trends in visitor and telephone restriction | | | | | | | | | |  |
| --- | --- | --- | --- | --- | --- | --- | --- | --- | --- | --- |
| Year | 2014 | 2015 | 2016 | 2017 | 2018 | 2019 | 2020 | 2021 | total | |
| With visitor restriction (n) | 27 | 49 | 64 | 68 | 57 | 55 | 51 | 63 | 434 | |
| Without visitor restriction (n) | 275 | 245 | 255 | 220 | 173 | 197 | 104 | 95 | 1564 | |
| All (n) | 302 | 294 | 319 | 288 | 230 | 252 | 155 | 158 | 1998 | |
| Visitor restriction ratio (%) | 8.90% | 16.70% | 20.10% | 23.60% | 24.80% | 21.80% | 32.90% | 39.90% | 21.70% | |
| With telephone restriction (n) | 69 | 93 | 95 | 98 | 81 | 78 | 53 | 64 | 631 | |
| Without telephone restriction (n) | 233 | 201 | 224 | 190 | 149 | 174 | 102 | 94 | 1367 | |
| All (n) | 302 | 294 | 319 | 288 | 230 | 252 | 155 | 158 | 1998 | |
| Telephone restriction ratio (%) | 22.80% | 31.60% | 29.80% | 34.00% | 35.20% | 31.00% | 34.20% | 40.50% | 31.60% | |

| Table S2. Association between demographic and clinical data and visitor restriction during the period ending December 31, 2019 | | | | |
| --- | --- | --- | --- | --- |
| Demographic and clinical data | With visitor restriction | Without visitor restriction | All | *p*-value |
| Age (mean, SD) | 45.40±14.70 | 50.96±17.40 | 49.90±17.05 | *3.3×10^-7^ |
| Hospitalization days (mean, SD) | 91.15±63.74 | 73.40±53.36 | 76.77±55.90 | *1.2×10^-7^ |
| Number of psychiatric hospitalizations (mean ± SD) | 3.66±4.17 | 3.99±4.19 | 3.93±4.19 | 0.04 |
|  |  | Visitor restriction ratio  n (%) | χ^2^ (df=1) | *p*-value |
| Sex | Female (n=1149) | 180(15.7%) | 25.96 | *3.4×10^-7^ |
|  | Male (n=536) | 140(26.1%) |  |  |
| Hospital admission type | Involuntary (n=1040) | 308(29.6%) | 199.34 | *2.9×10^-45^ |
|  | Voluntary (n=645) | 12(1.9%) |  |  |
| Seclusion during hospitalization | Yes (n=294) | 221(75.2%) | 730.61 | *6.6×10^-161^ |
|  | No (n=1391) | 99(7.1%) |  |  |
| Physical restraint during hospitalization | Yes (n=510) | 194(38.0%) | 172.49 | *2.1×10^-39^ |
|  | No (n=1175) | 126(10.7%) |  |  |
| As the level of significance, *p* < 5.7 × 10^−4^ was within the 5% significance level, based on the Bonferroni correction, and it was considered in the multiplicity of the tests.  **p* < 0.05, after the Bonferroni correction. | | | | |

| Table S3. Association between demographic and clinical data and telephone restriction during the period ending December 31, 2019 | | | | |
| --- | --- | --- | --- | --- |
| Demographic and clinical data | With telephone restriction | Without telephone restriction | All | *p*-value |
| Age (mean, SD) | 46.55±16.22 | 51.37±17.21 | 49.90±17.05 | *1.2×10^-7^ |
| Hospitalization days (mean, SD) | 89.02±62.91 | 71.39±51.64 | 76.77±55.90 | *2.5×10^-8^ |
| Number of psychiatric hospitalizations (mean ± SD) | 3.70±4.01 | 4.02±4.26 | 3.93±4.19 | 0.14 |
|  |  | Telephone restriction ratio  n (%) | χ^2^ (df=1) | *p*-value |
| Sex | Female (n=1149) | 310(27.0%) | 21.17 | *4.2×10^-6^ |
|  | Male (n=536) | 204(38.1%) |  |  |
| Hospital admission type | Involuntary (n=1040) | 497(47.8%) | 382.86 | *3.0×10^-85^ |
|  | Voluntary (n=645) | 17(2.6%) |  |  |
| Seclusion during hospitalization | Yes (n=294) | 262(89.1%) | 577.11 | *1.6×10^-127^ |
|  | No (n=1391) | 252(18.1%) |  |  |
| Physical restraint during hospitalization | Yes (n=510) | 318(62.4%) | 349.94 | *4.4×10^-78^ |
|  | No (n=1175) | 196(16.7%) |  |  |
| As the level of significance, *p* < 5.7 × 10^−4^ was within the 5% significance level, based on the Bonferroni correction, and it was considered in the multiplicity of the tests.  **p* < 0.05, after the Bonferroni correction. | | | | |

| Table S4. Association between psychiatric diagnosis and visitor restriction during the period ending December 31, 2019 | | | | |
| --- | --- | --- | --- | --- |
| Diagnosis (including co-morbid diagnoses) |  | Visitor restriction ratio  n (%) | χ^2^ (df=1) | *p*-value |
| Schizophrenia spectrum and other psychotic disorders | Yes (n=699) | 195(27.9%) | 61.59 | *4.2×10^-15^ |
|  | No (n=986) | 125(12.7%) |  |  |
| Bipolar and related disorders | Yes (n=130) | 37(28.5%) | 8.21 | 4.2×10^-3^ |
|  | No (n=1555) | 283(18.2%) |  |  |
| Bipolar 1 disorder manic episode | Yes (n=52) | 29(55.8%) | 47.18 | *6.5×10^-12^ |
|  | No (n=1633) | 291(17.8%) |  |  |
| Bipolar 1 disorder depressive episode | Yes (n=67) | 5(7.5%) | 6.03 | 0.01 |
|  | No (n=1618) | 315(19.5%) |  |  |
| Depressive disorders | Yes (n=380) | 31(8.2%) | 37.43 | *9.5×10^-10^ |
|  | No (n=1305) | 289(22.1%) |  |  |
| Anxiety disorders | Yes (n=46) | 3(6.5%) | 4.78 | 0.03 |
|  | No (n=1639) | 317(19.3%) |  |  |
| Obsessive–compulsive and related disorders | Yes (n=30) | 1(3.3%) | 4.87 | 0.03 |
|  | No (n=1655) | 319(18.9%) |  |  |
| Trauma and stressor-related disorders | Yes (n=24) | 0(0%) | 5.71 | 0.02 |
|  | No (n=1661) | 320(19.3%) |  |  |
| Dissociative disorders | Yes (n=77) | 12(15.6%) | 0.61 | 0.44 |
|  | No (n=1608) | 308(19.2%) |  |  |
| Somatic symptoms and related disorders | Yes (n=40) | 5(12.5%) | 1.12 | 0.29 |
|  | No (n=1645) | 315(19.1%) |  |  |
| Feeding and eating disorders | Yes (n=48) | 4(8.3%) | 3.65 | 0.06 |
|  | No (n=1637) | 316(19.3%) |  |  |
| Neurodevelopmental disorders | Yes (n=171) | 22(12.9%) | 4.64 | 0.03 |
|  | No (n=1514) | 298(19.7%) |  |  |
| Substance-related and addictive disorders | Yes (n=87) | 21(24.1%) | 1.58 | 0.21 |
|  | No (n=1598) | 299(18.7%) |  |  |
| Neurocognitive disorders | Yes (n=59) | 3(5.1%) | 7.69 | 0.01 |
|  | No (n=1626) | 317(19.5%) |  |  |
| Personality disorders | Yes (n=61) | 11(18.0%) | 0.04 | 0.85 |
|  | No (n=1624) | 309(19.0%) |  |  |
| As the level of significance, p < 5.7 × 10^−4^ was within the 5% significance level, based on the Bonferroni correction, and it was considered in the multiplicity of the tests.  **p* < 0.05, after the Bonferroni correction. | | | | |

| Table S5. Association between psychiatric diagnosis and telephone restriction during the period ending December 31, 2019 | | | | |  |
| --- | --- | --- | --- | --- | --- |
| Diagnosis (including co-morbid diagnoses) |  | Telephone restriction ratio  n (%) | χ^2^ (df=1) | *p*-value |  |
| Schizophrenia spectrum and other psychotic disorders | Yes (n=699) | 286(40.9%) | 61.08 | *5.5×10^-15^ |  |
|  | No (n=986) | 228(23.1%) |  |  |  |
| Bipolar and related disorders | Yes (n=130) | 47(36.2%) | 2.12 | 0.15 |  |
|  | No (n=1555) | 467(30.0%) |  |  |  |
| Bipolar 1 disorder manic episode | Yes (n=52) | 36(69.2%) | 37.96 | *7.2×10^-10^ |  |
|  | No (n=1633) | 478(29.3%) |  |  |  |
| Bipolar 1 disorder depressive episode | Yes (n=67) | 8(11.9%) | 11.34 | 7.5×10^-4^ |  |
|  | No (n=1618) | 506(31.3%) |  |  |  |
| Depressive disorders | Yes (n=380) | 55(14.5%) | 59.48 | *1.2×10^-14^ |  |
|  | No (n=1305) | 459(35.2%) |  |  |  |
| Anxiety disorders | Yes (n=46) | 6(13.0%) | 6.80 | 0.01 |  |
|  | No (n=1639) | 508(31.0%) |  |  |  |
| Obsessive–compulsive and related disorders | Yes (n=30) | 2(6.7%) | 8.19 | 4.2×10^-3^ |  |
|  | No (n=1655) | 512(30.9%) |  |  |  |
| Trauma and stressor-related disorders | Yes (n=24) | 1(4.2%) | 7.97 | 4.8×10^-3^ |  |
|  | No (n=1661) | 513(30.9%) |  |  |  |
| Dissociative disorders | Yes (n=77) | 16(20.8%) | 3.60 | 0.06 |  |
|  | No (n=1608) | 498(31.0%) |  |  |  |
| Somatic symptoms and related disorders | Yes (n=40) | 5(12.5%) | 6.27 | 0.01 |  |
|  | No (n=1645) | 509(30.9%) |  |  |  |
| Feeding and eating disorders | Yes (n=48) | 24(50.0%) | 8.86 | 2.9×10^-3^ |  |
|  | No (n=1637) | 490(29.9%) |  |  |  |
| Neurodevelopmental disorders | Yes (n=171) | 45(26.3%) | 1.58 | 0.21 |  |
|  | No (n=1514) | 469(31.0%) |  |  |  |
| Substance-related and addictive disorders | Yes (n=87) | 35(40.2%) | 4.09 | 0.04 |  |
|  | No (n=1598) | 479(30.0%) |  |  |  |
| Neurocognitive disorders | Yes (n=59) | 16(27.1%) | 0.33 | 0.57 |  |
|  | No (n=1626) | 498(30.6%) |  |  |  |
| Personality disorders | Yes (n=61) | 13(21.3%) | 2.52 | 0.11 |  |
|  | No (n=1624) | 501(30.8%) |  |  |  |
| As the level of significance, p < 5.7 × 10^−4^ was within the 5% significance level, based on the Bonferroni correction, and it was considered in the multiplicity of the tests.  **p* < 0.05, after the Bonferroni correction. | | | | |  |

| Table S6. The results of multivariable logistic regression analyses for visitor restriction excluding participants subject to seclusion and physical restraint | | | | |
| --- | --- | --- | --- | --- |
| Independent variables | Odds ratio | 95% CI | | *p*-value |
|  |  | Lower limit | Upper Limit |  |
| Age | 0.992 | 0.973 | 1.011 | 0.39 |
| Sex (female) | 0.902 | 0.445 | 1.832 | 0.78 |
| Hospitalization days | 1.001 | 0.996 | 1.006 | 0.64 |
| Involuntary admission | 4.440 | 2.063 | 9.556 | *1.4×10^-4^ |
| Schizophrenia spectrum and other psychotic disorders | 0.945 | 0.439 | 2.033 | 0.89 |
| Bipolar I disorder manic episode | 3.556 | 0.700 | 18.06 | 0.13 |
| Depressive disorders | 1.089 | 0.435 | 2.728 | 0.86 |
| The analysis included 1230 participants, excluding those who had been subjected to seclusion or physical restraint. Of these, 40 had been subject to visitor restrictions.  **p* < 0.05 | | | | |

| Table S7. The results of multivariable logistic regression analyses for telephone restriction excluding participants subject to seclusion and physical restraint | | | | |
| --- | --- | --- | --- | --- |
| Independent variables | Odds ratio | 95% CI | | *p*-value |
|  |  | Lower limit | Upper Limit |  |
| Age | 0.980 | 0.967 | 0.993 | *3.4×10^-3^ |
| Sex (female) | 1.055 | 0.628 | 1.771 | 0.84 |
| Hospitalization days | 1.005 | 1.001 | 1.008 | *5.8×10^-3^ |
| Involuntary admission | 14.63 | 6.95 | 30.79 | *1.6×10^-12^ |
| Schizophrenia spectrum and other psychotic disorders | 0.892 | 0.528 | 1.507 | 0.67 |
| Bipolar I disorder manic episode | 3.628 | 0.986 | 13.36 | 0.05 |
| Depressive disorders | 0.781 | 0.390 | 1.565 | 0.49 |
| The analysis included 1230 participants, excluding those who had been subjected to seclusion or physical restraint. Of these, 91 had been subject to telephone restrictions.  *：*p* < 0.05 | | | | |
